# Toxicity of Proton Therapy versus Photon Therapy on Salvage Re-Irradiation for Non-Small Cell Lung Cancer

**DOI:** 10.3390/life12020292

**Published:** 2022-02-16

**Authors:** Kyungmi Yang, Yang-Gun Suh, Hyunju Shin, Hongryull Pyo, Sung Ho Moon, Yong Chan Ahn, Dongryul Oh, Eunah Chung, Kwanghyun Jo, Jae Myoung Noh

**Affiliations:** 1 Department of Radiation Oncology, Samsung Medical Center, Sungkyunkwan University School of Medicine, Seoul 06351, Korea; kyungmi.yang@samsung.com (K.Y.); hyunjuhaha.shin@samsung.com (H.S.); hr.pyo@samsung.com (H.P.); ycahn.ahn@samsung.com (Y.C.A.); dongryul.oh@samsung.com (D.O.); eunah.chung@samsung.com (E.C.); kwanghyun.jo@samsung.com (K.J.); 2 Proton Therapy Center, Research Institute and Hospital, National Cancer Center, Goyang 10408, Korea; suhmd@ncc.re.kr (Y.-G.S.); shmoon@ncc.re.kr (S.H.M.)

**Keywords:** lung cancer, non-small cell lung cancer, radiotherapy, re-irradiation, locoregional recurrence, proton beam therapy, intensity-modulated radiation therapy, radiation-induced complication

## Abstract

This study evaluated the toxicity associated with radiation techniques on curative re-irradiation (re-RT) in patients with thoracic recurrence of non-small cell lung cancer (NSCLC). From 2011 to 2019, we retrospectively reviewed the data of 63 patients with salvage re-RT for in-field or marginal recurrence of NSCLC at two independent institutions. Re-RT techniques using X-ray beams and proton beam therapy (PBT) were also included. Re-RT had a 2-year overall survival (OS) and local progression-free survival of 48.0% and 52.0%, respectively. Fifteen patients experienced grade 3 or higher toxicity after re-RT. The complication rates were 18.2% (4/22) and 26.8% (11/41) in PBT patients and X-ray patients, respectively. Airway or esophageal fistulas occurred in seven patients (11.1%). Fistulas or severe airway obstruction occurred in patients with tumors adjacent to the proximal bronchial tree and esophagus, who underwent hypofractionated radiotherapy (RT) or concurrent chemotherapy, and with a higher dose exposure to the esophagus. In conclusion, salvage re-RT was feasible even in patients with local recurrence within the previous RT field. PBT showed similar survival outcomes and toxicity to those of other techniques. However, thoracic re-RT should be performed carefully considering tumor location and RT regimens such as the fraction size and concurrent chemotherapy.

## 1. Introduction

Lung cancer is the most commonly diagnosed cancer and the leading cause of cancer-related mortality worldwide [1]. Of all lung cancer patients, 85% are diagnosed with non-small cell lung cancer (NSCLC) [2]. The treatment options for NSCLC include surgery, radiotherapy (RT), chemotherapy, or multimodal therapy, depending on the clinical stage and the patient’s condition. RT combined with chemotherapy is the core treatment strategy for locally advanced NSCLC [3,4]. However, after curative treatment, 3–20% of patients experience thoracic recurrence despite the absence of distant metastasis [5]. As a treatment for locoregional recurrence, salvage surgery is recommended for patients with a history of RT. However, patients are usually diagnosed with advanced stages or inoperable conditions. In these cases, despite the high risk of treatment-related complications, salvage re-irradiation (re-RT) can be carefully considered [5,6,7].

Advancements in RT techniques, such as intensity-modulated radiation therapy (IMRT) and proton beam therapy (PBT), have led to the practice of thoracic re-RT in the clinical setting. In terms of clinical results, re-RT for locoregional recurrent NSCLC shows a 2-year overall survival (OS) of 30–40%, with a grade ≥3 lung toxicity rate of 0–20% [8]. PBT has less risk owing to its unique characteristic, the Bragg peak. However, its outcome is similar to that of conventional RT or IMRT, and some studies have even reported higher toxicity rates of >40% [9]. Previously, our study on salvage PBT including 37 patients who underwent re-RT showed a 2-year OS of 79.2% and a grade 3 or higher toxicity rate of 18.9% [5], which included out-field thoracic recurrences.

In this study, we aimed to compare the toxicity between PBT and RT techniques using X-ray as salvage re-RT for the in-field or marginal recurrence of NSCLC patients.

## 2. Materials and Methods

### 2.1. Patients

We retrospectively reviewed the medical records of 81 patients with locoregional recurrence of NSCLC who underwent salvage re-RT between January 2011 and December 2019 at the Samsung Medical Center and National Cancer Center, which are independent institutions with proton beam therapy (PBT) and X-ray radiotherapy (XRT) facilities. The patients were initially diagnosed with NSCLC and underwent RT with curative intent. The patterns of failure after initial RT were considered as the inclusion criteria and were defined as follows: in-field failure, wherein the geometric center of the recurrent mass is within the 50% isodose line, and marginal failure, wherein it is within 25–50% of the isodose line [5,8]. Moreover, patients who underwent re-RT with curative intent were included in the study. Patients who had undergone palliative re-RT, received a biologically effective radiation dose with an alpha/beta ratio of 10 Gy (BED10) <60 Gy or an equivalent dose at a fractionation of 2 Gy (EQD2) <50 Gy (*n* = 17), or adjuvant re-RT after salvage surgery were excluded (*n* = 1). Finally, 63 patients were included in the study. This study was approved by the Institutional Review Board (IRB) of Samsung Medical Center (No. 2021-01-019), and the requirement for informed consent was waived by the IRB because of the retrospective nature of the study.

### 2.2. Re-Irradiation

All patients underwent computed tomography (CT)-based simulations in the supine position. As target delineation for re-RT, the gross tumor volume included all suspicious malignant lesions, while the internal target volume was delineated based on the respiratory phases of four-dimensional CT. The clinical target volume (CTV) was expanded with a 5–7 mm margin from the ITV and was modified according to the anatomical boundary of the adjacent organs, if necessary. The planning target volume or block margin was determined based on the RT modalities: 5 mm for IMRT, stereotactic body radiotherapy (SBRT), and PBT; and 1.0–1.5 cm for three-dimensional conformal radiotherapy (3D-CRT). RT doses were administered in various fractionated schedules based on the treatment conditions, including the estimated risk of esophagitis: 60–70 Gy with 2–2.2 Gy per fraction for concurrent chemoradiotherapy, 50–66 Gy with 2–8 Gy per fraction for RT alone, and 48–60 Gy with 12–15 Gy per fraction for SBRT [3,5,10,11]. Generally, the dose fractionation schedules did not differ between XRT and PBT or between the two centers.

We also geometrically reviewed the re-RT plans for all patients. The centrality of the re-RT target was defined as follows: peripheral, distant from the organs at risk (OARs) such as the proximal bronchial tree, trachea, or esophagus; central, within 2 cm from the OARs; and ultracentral, abutted to the OARs. 

For dosimetric analyses, the doses for initial RT and re-RT were merged and calibrated based on the anatomy shown on simulation CT at re-RT using the RayStation treatment planning system (TPS) version 6.2.0.7 (RaySearch Laboratories, Stockholm, Sweden). The dose distribution from the initial RT was deformed according to the results of the simulation CT at re-RT using deformable image registration, and the final dose distribution from the initial and re-RT was calculated using the RayStation TPS. The radiation oncologists and two experienced medical physicists (E.C. and K.J.) confirmed that cumulative dosimetry worked properly for all patients.

### 2.3. Clinical Outcomes and Statistical Analysis

Treatment complications were scored and compared using the Radiation Therapy Oncology Group (RTOG) toxicity scoring criteria [12]. For comparison of the characteristics and dosimetric parameters, Fisher’s test for discrete variables and t-test or Mann–Whitney U-test for continuous variables were used. 

The OS and local progression-free survival (LPFS) from re-RT defined the length of time between the start of re-RT and events of death and radiological/clinical progression in the re-RT field (including death), and were calculated using the Kaplan–Meier method. The log-rank test for univariate analysis and Cox proportional hazard regression for multivariate analysis was performed based on the variables, with a *p* value of <0.1 from the univariate analysis.

The analyses were carried out in a hypothesis-generating approach and no correction for multiple testing was adopted. Statistical significance was set at *p* < 0.05. R 4.0.3 (R Development Core Team, Vienna, Austria, http://www.R-project.org, accessed on 15 August 2021) was used to perform all statistical analyses.

## 3. Results

### 3.1. Patients’ Characteristics

Of the 63 patients, 38 (60.3%) had in-field recurrence and 25 (39.7%) had marginal recurrence at re-RT. The median interval between the initial RT and re-RT was 15.2 (range: 3.2–44.6) months. None of the patients underwent additional thoracic RT during initial RT and re-RT. The characteristics of the 63 patients are shown in Table 1. The median age at NSCLC diagnosis was 63 years (range: 44–84 years). The majority of the patients were men (56, 88.9%), had a history of smoking (52, 82.5%), and had a histologic type of squamous cell carcinoma (41, 65.1%). Twelve patients (19.0%) had underlying chronic obstructive pulmonary disease. The median forced expiratory volume in the first second (FEV1) and the diffusing capacity of the lungs for carbon monoxide (DLCO) were 2.2 L and 72%, respectively, at re-RT. Concurrent chemotherapy was performed at re-RT in 21 patients (33.3%), XRT in 41 patients (65.1%), and PBT in 22 patients (34.9%). Further details of the initial RT are provided in Table S1. In comparison between the XRT and PBT groups, most characteristics were not significant except performance status, which was not a clinically meaningful difference.

Regarding the tumor location at re-RT, the central or ultracentral tumors in the trachea, proximal bronchi, and esophagus were not significantly different between XRT and PBT (*p* = 0.215, 0.648, and 0.712, respectively) (Table 2).

A dosimetric comparison of the re-RT plans was performed between the XRT and PBT groups (Table S2). At re-RT, the PBT group had a significantly lower lung dose, mean dose (*p* = 0.001), V20 (*p* = 0.016), and V5 (*p* = 0.001) than the XRT group. Furthermore, the cumulative results were similar; the lung dose and V50 of the esophagus (*p* = 0.034) were lower in the PBT group.

### 3.2. Clinical Outcomes

The median follow-up period was 19.7 (range: 5.0–102.7) months from the re-RT. The median interval from the initial RT to the re-RT was 15.4 (range: 3.2–44.6) months. The numbers of patients who died and developed local recurrence during the follow-up period were 38 (60.3%) and 25 (39.7%), respectively. The causes of death were cancer progression (23), treatment-related (7), and others (8). The treatment-related causes of death were fatal events during bronchoscopy (2) and fistulae of the esophagus, trachea, or bronchus (5), which were reported in the XRT group, and in only one patient in the PBT group.

The median LPFS from re-RT was 27.1 months, and the 2-year LPFS was 52.0%. Additionally, the OS with re-RT was 22.7 months, while the 2-year OS was 48.0%. No significant differences were observed in the LPFS and OS according to the re-RT technique. In the univariate analysis of LPFS, squamous cell carcinoma was more severe than the other histologic types (*p* = 0.030) (Table S3). Furthermore, patients with squamous type, DLCO of <60%, and CTV of ≥75 cc had significantly poorer OS (*p* = 0.004, 0.004, and 0.037, respectively) (Table S1). In the multivariate analysis of OS, the significant hazard ratios of squamous type and DLCO of <60% were 5.575 (95% confidence interval (CI): 1.221–25.456, *p* = 0.027) and 4.058 (95% CI: 1.014–16.322, *p* = 0.048), respectively.

### 3.3. Radiation-related Complications

**Figure 1:**
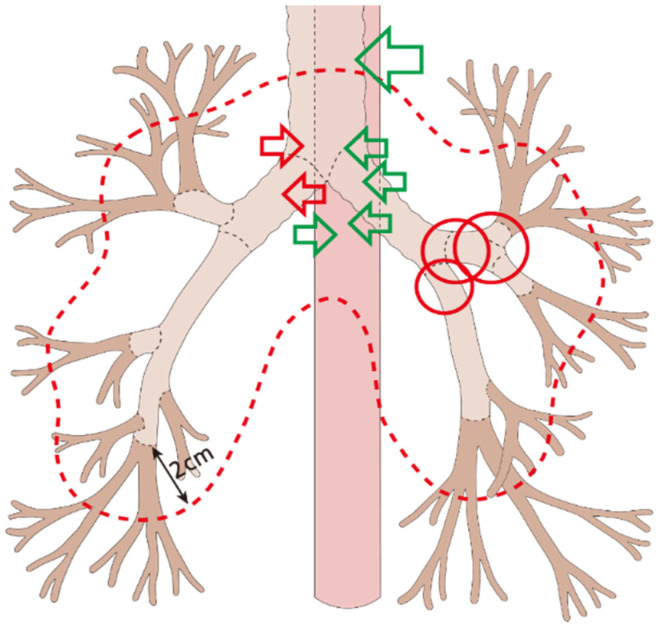
Location of fistula and obstruction after re-RT in the trachea, bronchi, and esophagus; broken line, within 2 cm of the proximal bronchial tree; red circles, bronchial obstruction; red arrows, bronchial fistula without esophageal damage; green arrows, esophageal fistula.

**Figure 2:**
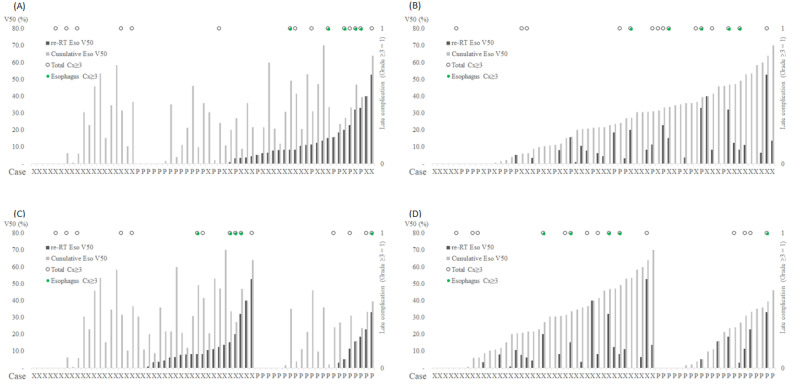
Individual distribution of grade ≥3 esophageal complication (*n* = 5) with V50 (%) of esophagus. (**A**) Sorted by V50 of esophagus at re-RT; (**B**) sorted by cumulative V50 of esophagus; (**C**) sorted by re-RT techniques and V50 of esophagus at re-RT; (**D**) sorted by re-RT techniques and cumulative V50 of esophagus; other figures with Dmax and Dmean of the esophagus, and Dmean and V20 of the lungs were attached as Figures S1–S4; Cx, complication; X, X-ray beam therapy; P, proton beam therapy.

The incidence of severe toxicity after re-RT, including RTOG grades 3–5, was assessed. First, acute toxicities of grades 3–5 were not reported. Meanwhile, 15 patients experienced severe RT-related complications, of whom 11 were in the XRT group and 4 were in the PBT group (26.8% vs. 18.2%, *p* = 0.647). The details of organs associated with the complications were as follows: lung (*n* = 7: two fistulas, three bronchial obstructions, one pneumothorax, and one radiation pneumonitis), esophagus (*n* = 5: all fistulas), heart (*n* = 2), and recurrent laryngeal nerve (*n* = 1). Of the 12 patients with lung or esophageal complications, 2 underwent PBT (bronchial obstruction: 1 and esophageal fistula: 1). The fistula and obstruction of the airway and esophagus are shown in Figure 1. Because these events rarely occurred, lung Dmean, V20, and esophageal Dmax, Dmean, and V50 in all cases are shown individually based on the type of complication and RT modality used (Figures S1–S4 and Figure 2). Esophageal complications occurred mainly in patients who received higher doses of Dmax (Figure S3) and larger volumes of V50 (Figure 2).

Additionally, a subgroup analysis was performed according to the lung and esophageal complications (Table S4), and the main factors are shown in Table 3. Patients who received a smaller number of fractions (*p* = 0.002) showed significantly higher lung complication rates. However, esophageal complications were more frequent in patients with ultracentral tumors in the esophagus and concurrent chemotherapy (*p* < 0.001 and 0.070, respectively). Additionally, factors associated with RT schedules including less BED_10_, more fractions, and small fraction size were significant, which seemed to be clinically related to concurrent chemoradiation therapy. Moreover, patients with severe complications had higher esophageal dosimetric parameters such as Dmax, Dmean, and V50 at re-RT and Dmax and Dmean in cumulative plans.

## 4. Discussion

This retrospective study at two institutions compared the toxicity between PBT and XRT for salvage re-RT in patients with in-field or marginal recurrence with a previous history of thoracic RT. Although the clinical outcome such as OS and LPFS were comparable to other studies, severe RT-related complications were not significantly different in RT techniques, 26.8% with the XRT and 18.2% with PBT. Although patients with out-field recurrence were excluded, the results of this study were comparable with those of other studies, as summarized in our previous study [5]. 

The administration of thoracic re-RT was carefully considered, as it can potentially cause severe complications. Frequently, patients had a maximum dose exposure at the OARs even before re-RT, while radiation-resistant recurrent tumors require high-dose RT of 60–66 Gy for curative treatment [13]. A study on PBT reported a grade ≥3 toxicity rate of 42% [14], while other studies showed tolerable toxicity rates. The risk factors for thoracic re-RT include the time interval between the initial RT and re-RT, tumor volume, tumor location, and dose exposure to critical organs [15]. Bronchial or esophageal fistulas (7/63, 11.1%) or airway obstruction (3/63, 4.8%) were reported in our study. No significant difference was observed between the time interval and tumor volume; the relationship between these two factors remains unclear in some previous studies [16]. Regarding tumor location, most events occurred in the adjacent proximal bronchi and esophagus (Figure 1). Chao et al. reported the risk of centrally located tumors in the region of thoracic re-RT [9]; McAvoy et al. also showed that the rates of cardiac and pulmonary toxicity increased in central tumors within 2 cm of the proximal bronchial tree [9,16]. Additionally, tumors in the esophagus are as important as those in the bronchial tree as broncho-esophageal fistulas commonly develop; one patient developed an esophageal fistula outside of the at-risk area within 2 cm of the proximal bronchial tree. This means that the location of tumors in the esophagus and proximal bronchial tree should be assessed to determine the need for re-RT. Therefore, centrally located or adjacent tumors should be considered relative contraindications for salvage re-RT. 

Another risk factor is dose exposure to critical organs. However, there are no definite guidelines for the dose constraints of thoracic RT. Most patients with esophageal fistulas received Dmax >65 Gy at re-RT and a cumulative Dmax >110 Gy. Additionally, the volume of irradiation, such as V50, showed a similar pattern. In 3D-CRT patients, it was difficult to control the OAR dose, and this seemed to cause more esophageal complications. Other studies also reported that a higher dose exposure or a higher radiation dose was related to the occurrence of esophageal complications [16]. Although the cut-off value could not be clearly established due to the rare occurrence of some adverse events, the esophageal dose in the re-RT plan and its cumulative dose are important factors that need to be evaluated. Volumetric parameters such as V20 or V5 and mean lung dose are usually used regardless of RT and RT status. Some studies have confirmed that a high volume or high dose of these parameters is a risk factor [16]. However, severe lung complications, such as bronchial fistula or obstruction, were not related to the usual parameters in this study. This means that critical injury during thoracic re-RT occurred in patients who received high doses of radiation in serial organs such as the bronchus and esophagus, and not the whole lungs. Therefore, thoracic re-RT was carefully performed in patients with tumors of the bronchial tree and esophagus with a dose limitation, although the constraints should be studied using a larger population.

Other factors require consideration to ensure safe re-RT. Salvage re-RT was performed heterogeneously, which included various RT fraction sizes [17] and techniques. SBRT has been considered in patients with small and localized tumors in the peripheral lung [18,19]. A hypofractionated schedule has been commonly administered, which was 39% in a multi-institutional study on PBT [17]. These studies suggest that hypofractionated schedules are safe and effective. However, these are inconclusive because the cases were very limited in terms of tumor extent and location. In our study, two cases of lung complications were reported in five patients who underwent SBRT as re-RT. Furthermore, some patients received concurrent chemotherapy [13]. One-third of our patients also underwent concurrent chemotherapy. However, the benefits of concurrent chemotherapy have not been confirmed for salvage re-RT. In this study, most patients with esophageal fistulas received concurrent chemotherapy at re-RT, although the *p*-value was 0.070. A multi-institutional prospective study also reported higher treatment toxicities with concurrent chemotherapy (53% vs. 16%, *p* = 0.003) [9]. Therefore, at re-RT, all settings, including RT regimens or concurrent chemotherapy, should be carefully evaluated.

Regarding the choice of re-RT technique, it remains uncertain whether PBT has better clinical outcomes, which has only been reported in a few studies with relatively small sample sizes [5,8,9,13,17]. Generally, PBT is associated with similar or higher treatment toxicity [5,20,21,22]. However, PBT exhibits strong physical and biological properties. PBT plans can spare relatively low-dose areas using the Bragg peak compared with IMRT. Hypofractionated RT may be a safe re-RT technique for patients with early recurrent stages; those with poor lung function or underlying lung disease might benefit from this technique [23]. In this study, lung complications were mostly observed in XRT patients, and most of the dosimetric lung parameters were better in the PBT group. Furthermore, the relative biological effectiveness of proton beams is approximately 1.1, which is still promising for increasing local control under the same disease conditions in terms of radioresistance [24]. We believe that PBT would be beneficial in some selected cases, but further studies are needed to confirm this.

This study had some limitations. Despite the collaboration between the two institutions, this study was retrospective in nature. Furthermore, the study population was relatively small, which was similar to that of other re-RT studies. Extremely rare toxicity-related events occurred in this study, which cannot be used for specific analyses. Unfortunately, the initial and salvage treatments were heterogeneous. Hence, a well-organized, prospective, larger-sized study is needed to determine the effectiveness and safety of specific RT techniques for re-RT.

## 5. Conclusions

Curative re-RT was feasible in patients with thoracic recurrence of NSCLC. PBT had the same survival outcomes and similar toxicity as those of X-ray techniques. In some patients with tumors abutting the bronchus and esophagus, severe complications such as fistulas were observed. Therefore, the OAR doses and tumor location, and RT regimens including the fraction size and concurrent chemotherapy, must be considered when planning thoracic re-RT.

## Figures and Tables

**Table 1:** Patients’ characteristics and comparison between patient groups according to re-RT modality.

Variables	Total (*N* = 63)	XRT (*n* = 41)	PBT (*n* = 22)	*p*-Value
Age, years				
Median (range)	63 (44–84)	62 (44–80)	66 (44–84)	0.163
Sex				
Male	56 (88.9%)	35 (85.4%)	21 (95.5%)	0.405
Female	7 (11.1%)	6 (14.6%)	1 (4.5%)	
Smoking				
Non-smoker	11 (17.5%)	8 (19.5%)	3 (13.6%)	0.733
Current or ex-moker	52 (82.5%)	33 (80.5%)	19 (86.4%)	
Histology				
SQ	41 (65.1%)	27 (65.9%)	14 (63.6%)	0.519
AD	20 (31.7%)	12 (29.3%)	8 (36.4%)	
Others or NOS	2 (3.2%)	2 (4.9%)	-	
CCI				
Median (range)	4 (1–7)	3 (1–6)	4 (1–7)	0.287
Underlying COPD				
No	51 (81.0%)	35 (85.4%)	16 (72.7%)	0.314
Yes	12 (19.0%)	6 (14.6%)	6 (27.3%)	
Re-RT interval, months				
Median (range)	15.2 (3.2–44.6)	15.8 (3.2–44.6)	14.0 (5.8–28.9)	0.096
ECOG at re-RT				
0–1	60 (95.2%)	41 (100%)	19 (86.4%)	0.039
2	3 (4.8%)	-	3 (13.6%)	
FEV1, L				
Median (range)	2.2 (1.2–3.8)	2.2 (1.2–3.8)	2.3 (1.5–2.9)	0.567
DLCO, %				
Median (range)	72 (25–118)	72 (25–118)	67 (25–118)	0.666
Clinical stage at re-RT				
I-II	30 (47.6%)	18 (43.9%)	12 (54.5%)	0.428
III	33 (52.4%)	23 (56.1%)	10 (45.5%)	
Recurrence at re-RT				
1st	56 (88.9%)	37 (90.2%)	19 (86.4%)	0.687
2nd or more	7 (11.1%)	4 (9.3%)	3 (13.6%)	
CCRT				
No	42 (66.7%)	26 (63.4%)	16 (72.7%)	0.578
Yes	21 (33.3%)	15 (36.6%)	6 (27.3%)	
Re-RT technique				
XRT-3D	23 (36.5%)	23 (56.1%)	-	-
XRT-IMRT	13 (20.6%)	13 (31.7%)	-	
XRT-SBRT	5 (7.9%)	5 (12.2%)	-	
PBT-3DPT	3 (4.8%)	-	3 (13.6%)	
PBT-IMPT	19 (30.2%)	-	19 (86.4%)	

Abbreviations: XRT, X-ray beam therapy; PBT, proton beam therapy; SQ, squamous cell carcinoma; AD, adenocarcinoma; NOS, not specified; CCI, Charlson comorbidity index; COPD, chronic obstructive pulmonary disease; RT, radiotherapy; ECOG, the Eastern Cooperative Oncology Group performance status; FEV1, the first second of forced expiration; DLCO, diffusing capacity of lung for carbon monoxide; CCRT, concurrent chemoradiation therapy; IMRT, intensity-modulated radiotherapy; SBRT, stereotactic body radiotherapy; 3DPT, three-dimensional proton therapy; IMPT, intensity-modulated radiotherapy.

**Table 2:** Re-RT target centrality based on the location of tumors in the trachea, proximal bronchi, and esophagus.

	XRT (*n* = 41)			PBT (*n* = 22)			*p*-Value
	Peripheral	Central	Ultracentral	Peripheral	Central	Ultracentral	
Trachea	22 (53.7%)	3 (7.3%)	16 (39.0%)	15 (68.2%)	3 (13.6%)	4 (18.2%)	0.215
Proximal bronchi	22 (53.7%)	7 (17.1%)	12 (29.3%)	12 (54.5%)	2 (9.1%)	8 (36.4%)	0.648
Esophagus	26 (63.4%)	9 (22.0%)	6 (14.6%)	13 (59.1%)	4 (18.2%)	5 (22.7%)	0.712

Abbreviations: XRT, X-ray beam therapy; PBT, proton beam therapy.

**Table 3:** Pulmonary and esophageal toxicities according to tumor location and dosimetric parameters.

	Lung			Esophagus		
Variables	Grades 0–2	Grades 3–5	*p*-Value	Grades 0–2	Grades 3–5	*p*-Value
Target—trachea location			0.534			1
Non-ultracentral	37 (86.0%)	6 (14.0%)		40 (93.0%)	3 (7.0%)	
Ultracentral	19 (95.0%)	1 (5.0%)		18 (90.0%)	2 (10.0%)	
Target—bronchus location			0.271			0.055
Non-ultracentral	40 (93.0%)	3 (7.0%)		42 (97.7%)	1 (2.3%)	
Ultracentral	16 (80.0%)	4 (20.0%)		16 (80.0%)	4 (20.0%)	
Target—esophagus location			1			<0.001
Non-ultracentral	46 (88.5%)	6 (11.5%)		52 (100%)	-	
Ultracentral	10 (90.9%)	1 (9.1%)		6 (54.5%)	5 (45.5%)	
CCRT at re-RT			0.119			0.07
No	35 (83.3%)	7 (16.7%)		41 (97.6%)	1 (2.4%)	
Yes	21 (100%)	-		17 (81.0%)	4 (19.0%)	
Re-RT technique			0.427			0.194
PBT	21 (95.5%)	1 (4.5%)		21 (95.5%)	1 (4.5%)	0.81
XRT	35 (85.4%)	6 (14.6%)		37 (90.2%)	4 (9.8%)	
rCTV, cc	81.4 ± 158.6	97.0 ± 92.3	0.801	87.3 ± 157.7	34.6 ± 41.4	0.074
BED_10_ at re-RT, Gy	85.3 ± 16.3	100.3 ± 34.1	0.292	87.6 ± 19.9	79.9 ± 2.3	0.009
Re-RT fractions	22.0 ± 9.5	14.0 ± 7.2	0.002	20.3 ± 9.4	30.8 ± 6.1	0.017
Re-RT fraction size, Gy	3.8 ± 3.0	6.7 ± 5.7	0.234	4.3 ± 3.5	2.2 ± 4.5	<0.001
Cumulative BED_10,_ Gy	164.4 ± 31.8	175.4 ± 28.6	0.387	167.6 ± 31.9	143.3 ± 11.7	0.098
Lung, Dmean, Gy	4.5 ± 2.9	6.4 ± 4.1	0.115			
Lung, V20, %	7.5 ± 5.4	10.4 ± 8.1	0.22			
Lung, V5, %	19.0 ± 13.0	24.7 ± 14.1	0.283			
Lung, cumulative Dmean, Gy	14.1 ± 6.2	15.5 ± 4.8	0.682			
Lung, cumulative V20, %	22.8 ± 12.2	24.8 ± 9.5	0.635			
Lung, cumulative V5, %	46.6 ± 19.3	50.3 ± 18.3	0.865			
Esophagus, Dmax, Gy				41.7 ± 23.8	67.2 ± 2.5	<0.001
Esophagus, Dmean, Gy				8.6 ± 8.1	19.0 ± 7.4	0.008
Esophagus, V50, %				4.8 ± 9.7	21.8 ± 10.7	<0.001
Esophagus, cumulative Dmax, Gy				83.6 ± 33.3	123.5 ± 10.4	<0.001
Esophagus, cumulative Dmean, Gy				25.0 ± 14.4	42.2 ± 11.3	0.012
Esophagus, cumulative V50, %				22.3 ± 19.4	39.4 ± 9.2	0.057

Abbreviations: RT, radiotherapy; CCRT, concurrent chemoradiation therapy; rCTV, clinical target volume at re-RT; BED_10_, biologically effective dose with alpha-beta ratio of 10 Gy; PBT, proton beam therapy. Numbers with underline: *p* < 0.05.

## Data Availability

Not applicable.

## Supplementary Materials

The following supporting information can be downloaded at https://www.mdpi.com/article/10.3390/life12020292/s1, Figures S1–S4: Individual distribution of grade ≥3 complications; Table S1: Details of initial RT; Table S2: Dosimetry parameters and comparison between the patient groups according to the re-RT modality; Table S3: Univariate analysis; Table S4: Pulmonary and esophageal toxicities according to patient characteristics and dosimetric parameters.

## References

[1] Bray F., Ferlay J., Soerjomataram I., Siegel R.L., Torre L.A., Jemal A. (2018). Global cancer statistics 2018: GLOBOCAN estimates of incidence and mortality worldwide for 36 cancers in 185 countries. CA Cancer J. Clin..

[2] Travis W.D., Brambilla E., Nicholson A.G., Yatabe Y., Austin J.H.M., Beasley M.B., Chirieac L.R., Dacic S., Duhig E., Flieder D.B. (2015). The 2015 World Health Organization Classification of Lung Tumors: Impact of Genetic, Clinical and Radiologic Advances Since the 2004 Classification. J. Thorac. Oncol..

[3] Noh J.M., Ahn Y.C., Lee H., Pyo H., Kim B., Oh D., Park H., Lee E., Park K., Ahn J.S. (2015). Definitive Bimodality Concurrent Chemoradiotherapy in Patients with Inoperable N2-positive Stage IIIA Non-small Cell Lung Cancer. Cancer Res. Treat..

[4] Noh J.M., Kim J.M., Ahn Y.C., Pyo H., Kim B., Oh D., Ju S.G., Kim J.S., Shin J.S., Hong C.S. (2016). Effect of Radiation Therapy Techniques on Outcome in N3-positive IIIB Non-small Cell Lung Cancer Treated with Concurrent Chemoradiotherapy. Cancer Res. Treat..

[5] Shin H., Noh J.M., Pyo H., Ahn Y.C., Oh D. (2021). Salvage proton beam therapy for locoregional recurrence of non-small cell lung cancer. Radiat. Oncol. J..

[6] Ebara T., Tanio N., Etoh T., Shichi I., Honda A., Nakajima N. (2007). Palliative re-irradiation for in-field recurrence after definitive radiotherapy in patients with primary lung cancer. Anticancer Res..

[7] Brooks E.D., Sun B., Feng L., Verma V., Zhao L., Gomez D.R., Liao Z., Jeter M., O’Reilly M., Welsh J.W. (2018). Association of Long-term Outcomes and Survival With Multidisciplinary Salvage Treatment for Local and Regional Recurrence After Stereotactic Ablative Radiotherapy for Early-Stage Lung Cancer. JAMA Netw. Open.

[8] McAvoy S., Ciura K., Wei C., Rineer J., Liao Z., Chang J.Y., Palmer M.B., Cox J.D., Komaki R., Gomez D.R. (2014). Definitive reirradiation for locoregionally recurrent non-small cell lung cancer with proton beam therapy or intensity modulated radiation therapy: Predictors of high-grade toxicity and survival outcomes. Int. J. Radiat. Oncol. Biol. Phys..

[9] Chao H.H., Berman A.T., Simone C.B., Ciunci C., Gabriel P., Lin H., Both S., Langer C., Lelionis K., Rengan R. (2017). Multi-Institutional Prospective Study of Reirradiation with Proton Beam Radiotherapy for Locoregionally Recurrent Non-Small Cell Lung Cancer. J. Thorac. Oncol..

[10] Cho W.K., Noh J.M., Ahn Y.C., Oh D., Pyo H. (2016). Radiation Therapy Alone in cT1-3N0 Non-small Cell Lung Cancer Patients Who Are Unfit for Surgical Resection or Stereotactic Radiation Therapy: Comparison of Risk-Adaptive Dose Schedules. Cancer Res. Treat..

[11] Lee S.U., Moon S.H., Cho K.H., Pyo H.R., Kim J.Y., Kim D.Y., Kim T.H., Suh Y.G., Kim Y.J. (2016). Ablative dose proton beam therapy for stage I and recurrent non-small cell lung carcinomas: Ablative dose PBT for NSCLC. Strahlenther. Onkol..

[12] Cox J.D., Stetz J., Pajak T.F. (1995). Toxicity criteria of the Radiation Therapy Oncology Group (RTOG) and the European Organization for Research and Treatment of Cancer (EORTC). Int. J. Radiat. Oncol. Biol. Phys..

[13] Ho J.C., Nguyen Q.N., Li H., Allen P.K., Zhang X., Liao Z., Zhu X.R., Gomez D., Lin S.H., Gillin M. (2018). Reirradiation of thoracic cancers with intensity modulated proton therapy. Pract. Radiat. Oncol..

[14] Vyfhuis M.A.L., Rice S., Remick J., Mossahebi S., Badiyan S., Mohindra P., Simone C.B. (2018). Reirradiation for locoregionally recurrent non-small cell lung cancer. J. Thorac. Dis..

[15] Chao H.H., Berman A.T. (2018). Proton therapy for thoracic reirradiation of non-small cell lung cancer. Transl. Lung Cancer Res..

[16] McAvoy S.A., Ciura K.T., Rineer J.M., Allen P.K., Liao Z., Chang J.Y., Palmer M.B., Cox J.D., Komaki R., Gomez D.R. (2013). Feasibility of proton beam therapy for reirradiation of locoregionally recurrent non-small cell lung cancer. Radiother. Oncol..

[17] Badiyan S.N., Rutenberg M.S., Hoppe B.S., Mohindra P., Larson G., Hartsell W.F., Tsai H., Zeng J., Rengan R., Glass E. (2019). Clinical Outcomes of Patients With Recurrent Lung Cancer Reirradiated With Proton Therapy on the Proton Collaborative Group and University of Florida Proton Therapy Institute Prospective Registry Studies. Pract. Radiat. Oncol..

[18] Maranzano E., Draghini L., Anselmo P., Casale M., Arcidiacono F., Chirico L., Italiani M., Trippa F. (2016). Lung reirradiation with stereotactic body radiotherapy. J. Radiosurg. SBRT.

[19] Ogawa Y., Shibamoto Y., Hashizume C., Kondo T., Iwata H., Tomita N., Ogino H. (2018). Repeat stereotactic body radiotherapy (SBRT) for local recurrence of non-small cell lung cancer and lung metastasis after first SBRT. Radiat. Oncol..

[20] Hong J.H., Kim Y.-S., Lee S.-W., Lee S.J., Kang J.H., Hong S.H., Hong J.-Y., Cheon G. (2019). High-dose thoracic re-irradiation of lung cancer using highly conformal radiotherapy is effective with acceptable toxicity. Cancer Res. Treat. Off. J. Korean Cancer Assoc..

[21] Griffioen G.H., Dahele M., de Haan P.F., van de Ven P.M., Slotman B.J., Senan S. (2014). High-dose, conventionally fractionated thoracic reirradiation for lung tumors. Lung Cancer.

[22] Trovo M., Minatel E., Durofil E., Polesel J., Avanzo M., Baresic T., Bearz A., Del Conte A., Franchin G., Gobitti C. (2014). Stereotactic body radiation therapy for re-irradiation of persistent or recurrent non-small cell lung cancer. Int. J. Radiat. Oncol. Biol. Phys..

[23] Kim H., Pyo H., Noh J.M., Lee W., Park B., Park H.Y., Yoo H. (2019). Preliminary result of definitive radiotherapy in patients with non-small cell lung cancer who have underlying idiopathic pulmonary fibrosis: Comparison between X-ray and proton therapy. Radiat. Oncol..

[24] Paganetti H., Niemierko A., Ancukiewicz M., Gerweck L.E., Goitein M., Loeffler J.S., Suit H.D. (2002). Relative biological effectiveness (RBE) values for proton beam therapy. Int. J. Radiat. Oncol. Biol. Phys..

