# Supplementary material for: Toxicity of Proton Therapy versus Photon Therapy on Salvage Re-Irradiation for Non-Small Cell Lung Cancer
Source: Life (Basel). 2022 Feb 16;12(2):292. doi: 10.3390/life12020292 (PMC8876714; doi:10.3390/life12020292)
Supplement: Supplementary file 1 [file life-12-00292-s001.zip › Supplementary figures.pptx]

## Slide 1
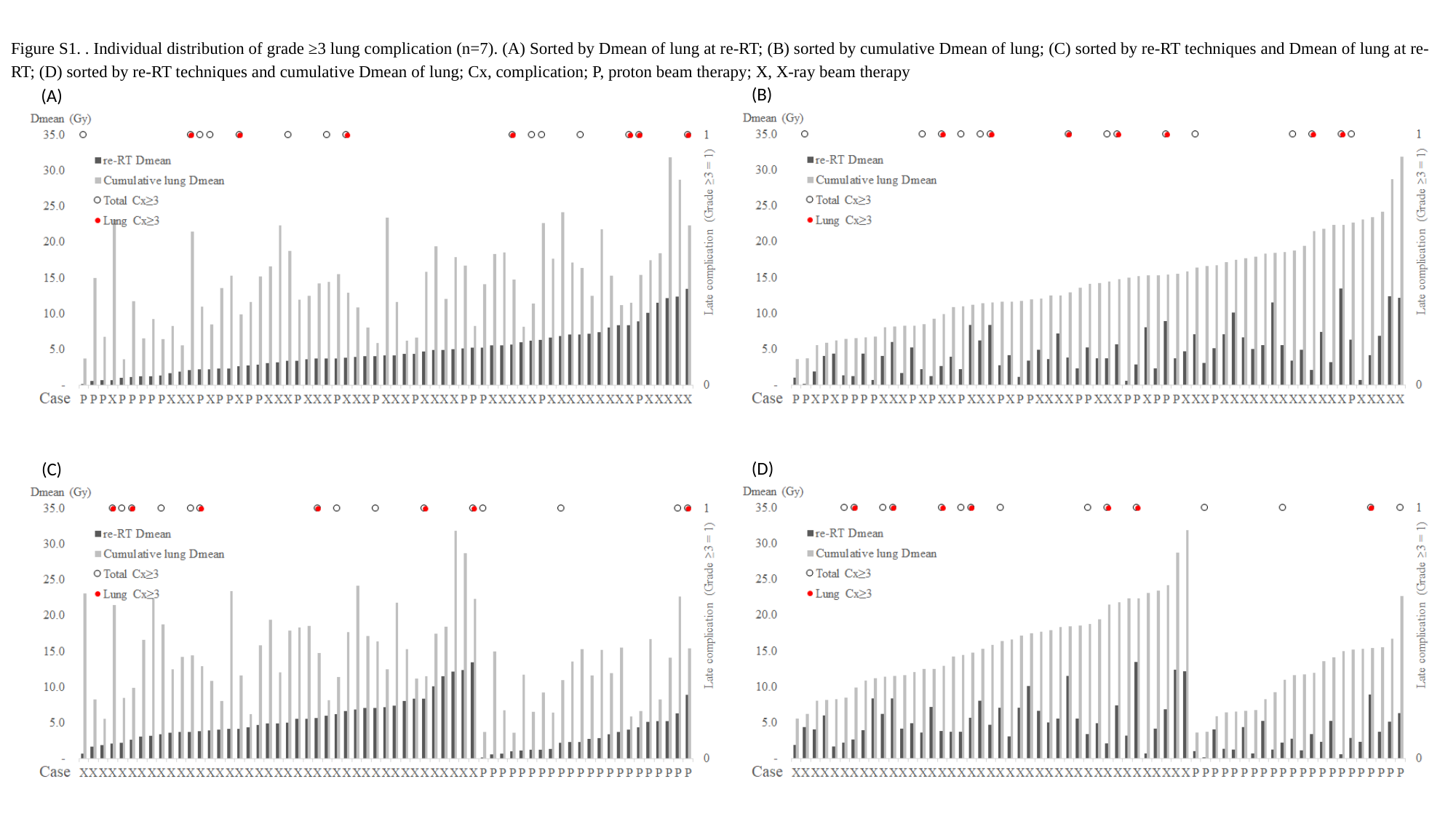

# Figure S1. . Individual distribution of grade ≥3 lung complication (n=7). (A) Sorted by Dmean of lung at re-RT; (B) sorted by cumulative Dmean of lung; (C) sorted by re-RT techniques and Dmean of lung at re-RT; (D) sorted by re-RT techniques and cumulative Dmean of lung; Cx, complication; P, proton beam therapy; X, X-ray beam therapy
(B)
(A)
(D)
(C)

## Slide 2
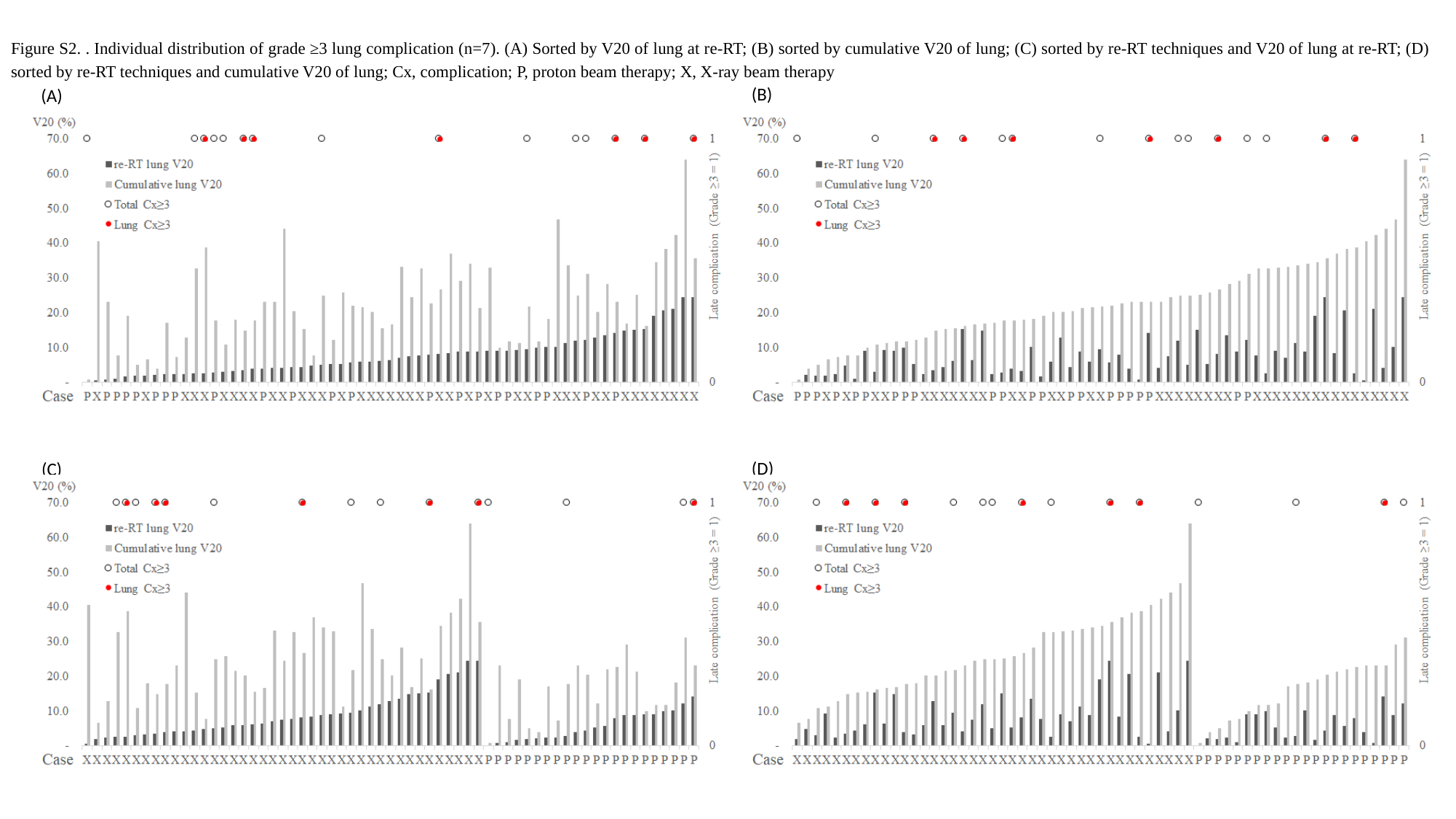

# Figure S2. . Individual distribution of grade ≥3 lung complication (n=7). (A) Sorted by V20 of lung at re-RT; (B) sorted by cumulative V20 of lung; (C) sorted by re-RT techniques and V20 of lung at re-RT; (D) sorted by re-RT techniques and cumulative V20 of lung; Cx, complication; P, proton beam therapy; X, X-ray beam therapy
(B)
(A)
(D)
(C)

## Slide 3
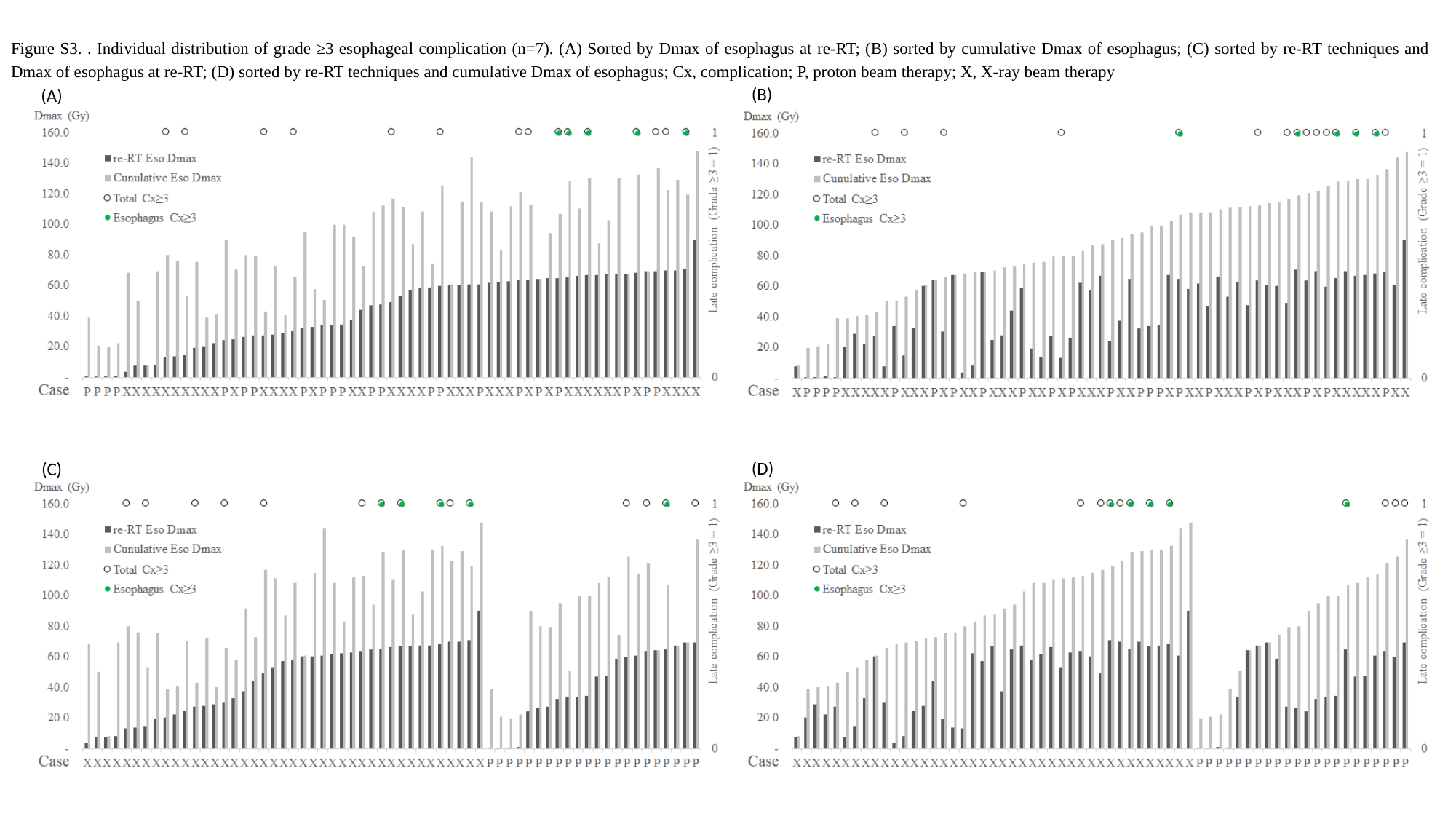

# Figure S3. . Individual distribution of grade ≥3 esophageal complication (n=7). (A) Sorted by Dmax of esophagus at re-RT; (B) sorted by cumulative Dmax of esophagus; (C) sorted by re-RT techniques and Dmax of esophagus at re-RT; (D) sorted by re-RT techniques and cumulative Dmax of esophagus; Cx, complication; P, proton beam therapy; X, X-ray beam therapy
(B)
(A)
(D)
(C)

## Slide 4
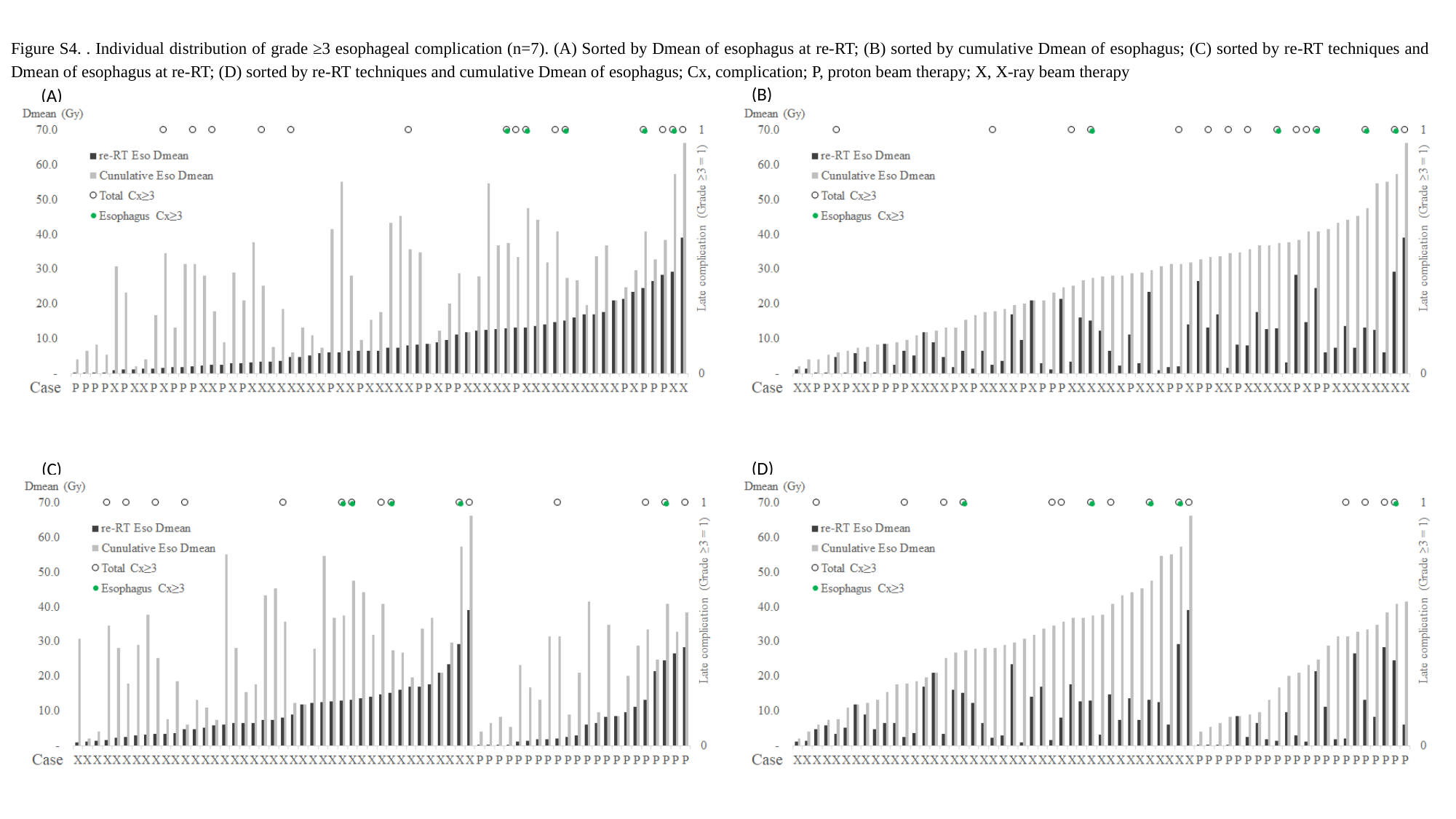

# Figure S4. . Individual distribution of grade ≥3 esophageal complication (n=7). (A) Sorted by Dmean of esophagus at re-RT; (B) sorted by cumulative Dmean of esophagus; (C) sorted by re-RT techniques and Dmean of esophagus at re-RT; (D) sorted by re-RT techniques and cumulative Dmean of esophagus; Cx, complication; P, proton beam therapy; X, X-ray beam therapy
(B)
(A)
(D)
(C)
