# Supplementary material for: Toxicity of Proton Therapy versus Photon Therapy on Salvage Re-Irradiation for Non-Small Cell Lung Cancer
Source: Life (Basel). 2022 Feb 16;12(2):292. doi: 10.3390/life12020292 (PMC8876714; doi:10.3390/life12020292)
Supplement: Supplementary file 1 [file life-12-00292-s001.zip › Supplementary tables.pdf]

Table S1. Details of initial RT

Variables	Total (N=63)	XRT (n=41)	PBT (n=22)	p-value
**ECOG**				
0-1	59 (93.7%)	40 (97.6%)	19 (86.4%)	0.118
2-3	4 (6.3%)	1 (2.4%)	3 (13.6%)	
**FEV1, L**				
Median (range)	2.5 (0.8–3.7)	2.5 (0.8–3.6)	2.6 (1.2–3.7)	0.974
**DLCO, %**				
Median (range)	78 (41–135)	86 (41–135)	72.5 (44–120)	0.050
**Clinical stage**				
I–II	13 (20.6%)	8 (19.5%)	5 (22.7%)	0.755
III–IVa	50 (79.4%)	33 (80.5%)	17 (79.3%)	
**Recurrence at initial RT**				
0	58 (92.1%)	38 (92.7%)	29 (90.9%)	0.624
1st	4 (6.3%)	2 (4.9%)	2 (9.1%)	
2nd or more	1 (1.6%)	1 (2.4%)	-	
**Aim of initial RT**				
Neoadjuvant	9 (14.3%)	7 (17.1%)	2 (9.1%)	0.271
Neoadjuvant + adjuvant	5 (7.9%)	4 (9.8%)	1 (4.5%)	
Definitive	39 (61.9%)	22 (53.7%)	17 (77.3%)	
Adjuvant	5 (7.9%)	5 (12.2%)	-	
Salvage	5 (7.9%)	3 (7.3%)	2 (9.1%)	
**CCRT**				
No	20 (31.7%)	10 (24.4%)	10 (45.5%)	0.099
Yes	43 (68.3%)	31 (75.6%)	12 (54.5%)	
**Initial RT technique**				
3D	34 (54.0%)	26 (63.4%)	8 (36.4%)	0.023
IMRT	21 (33.3%)	13 (31.7%)	8 (36.4%)	
SBRT	2 (3.2%)	1 (2.4%)	1 (4.5%)	
3DPT	2 (3.2%)	-	2 (9.1%)	
IMPT	4 (6.3%)	1 (2.4%)	3 (13.6%)	

Table S2. Dosimetry parameters and comparison between the patient groups according to the re-RT modality

Variables	Total (N=63)	XRT (N=41)	PBT (N=22)	p-value
**Initial RT**				
**BED_{10}, Gy**	78.7 ± 22.3	76.3 ± 23.7	83.1 ± 19.1	0.225
**iCTV, cc**	184.6 ± 127.5	181.3 ± 135.5	190.7 ± 113.8	0.642
**Lung**				
Dmean, Gy	9.7 ± 4.89	10.4 ± 5.1	8.3 ± 4.2	0.096
V20, %	17.7 ± 13.6	17.8 ± 10.1	17.6 ± 18.9	0.961
V5, %	39.4 ± 17.9	44.0 ± 17.5	30.7 ± 15.5	0.004
**Esophagus**				
Dmax, Gy	50.7 ± 19.2	51.0 ± 18.4	50.1 ± 21.0	0.854
Dmean, Gy	17.6 ± 11.6	19.3 ± 12.2	14.2 ± 9.7	0.096
V50, %	12.7 ± 16.4	14.8 ± 17.6	8.9 ± 13.4	0.172
**Heart**				
Dmax, Gy	51.4 ± 19.3	51.7 ± 16.8	50.9 ± 23.5	0.873
Dmean, Gy	90.3 ± 74.7	10.0 ± 7.8	7.3 ± 6.6	0.184
V25, %	14.0 ± 14.5	16.8 ± 15.7	8.9 ± 10.4	0.022
**Re-RT**				
**BED_{10}, Gy**	87.0 ± 9.6	87.6 ± 21.1	85.6 ± 15.6	0.709
**rCTV, cc**	83.1 ± 152.2	56.1 ± 51.4	133.6 ± 243.4	0.154
**Lung**				
Dmean, Gy	4.7 ± 3.0	5.6 ± 3.1	3.0 ± 2.2	0.001
V20, %	7.8 ± 5.8	9.0 ± 6.2	5.7 ± 4.1	0.016
V5, %	22.9 ± 28.8	29.6 ± 33.5	10.2 ± 6.6	0.001

**Esophagus**				
Dmax, Gy	43.8 ± 23.8	45.6 ± 23.6	40.4 ± 24.5	0.413
Dmean, Gy	9.4 ±8.5	10.2 ± 8.1	8.1 ± 9.2	0.355
V50, %	6.2 ± 10.8	6.7 ± 11.5	5.3 ± 9.7	0.616
**Heart**				
Dmax, Gy	33.5 ± 28.0	34.9 ± 27.4	30.8 ± 29.5	0.586
Dmean, Gy	3.7 ± 5.2	3.8 ± 4.7	3.5 ± 6.2	0.826
V25, %	4.8 ± 8.6	5.1 ± 9.3	4.3 ± 7.2	0.753
**Cumulative calculation**				
**BED_{10}, Gy**	165.7 ± 31.5	164.0 ± 33.8	168.8 ± 27.0	0.565
**Lung**				
Dmean, Gy	14.3 ± 6.0	15.9 ± 6.0	11.2 ± 4.9	0.003
V20, %	23.0 ± 11.9	26.6 ± 12.0	16.3 ± 8.3	0.001
V5, %	47.0 ± 19.0	54.1 ± 17.6	33.8 ± 14.1	<0.001
**Esophagus**				
Dmax, Gy	86.6 ± 33.9	89.5 ± 33.4	81.2 ± 35.0	0.360
Dmean, Gy	26.4 ± 14.9	28.7 ± 15.7	22.0 ± 12.5	0.088
V50, %	23.5 ± 19.3	27.3 ± 20.2	16.5 ± 15.4	0.034
V70, %	10.4 ± 13.2	11.8 ± 14.1	7.8 ± 11.2	0.255
**Heart**				
Dmax, Gy	80.7 ± 36.2	82.4 ± 30.3	77.5 ± 45.9	0.657
Dmean, Gy	14.3 ± 14.1	16.0 ± 15.9	11.1 ± 9.6	0.127
V25, %	19.1 ± 16.8	21.7 ± 17.8	14.2 ± 13.9	0.093
V50, %	7.6 ± 10.8	8.6 ± 12.2	5.7 ± 7.2	0.235

Abbreviations: XRT, X-ray beam therapy; PBT, proton beam therapy; RT, radiotherapy; BED10, biologically effective dose with an alpha-beta ratio of 10 Gy; iCTV, clinical target volume at initial RT; rCTV, clinical target volume at re-RT.

Table S3. Univariate analysis

		Local progression-free survival		Overall survival	
**Variables**	n	2 years	p-value	2 years	p-value
**Total**	63	51.6%		46.8%	
**Age at re-RT**			0.073		0.669
<65 years	33	57.7%		51.6%	
≥65 years	30	45.4%		38.2%	
**Sex**			0.520		0.194
Female	7	66.7%		44.1%	
Male	56	49.6%		68.6%	
**Smoking**			0.414		0.464
Non-smoker	11	72.9%		60.6%	
Current or Ex-smoker	52	47.0%		44.2%	
**ECOG at re-RT**			0.694		0.619
0-1	60	49.5%		46.1%	
2	3	100%		66.7%	
**CCI**			0.160		0.052
1–2	16	64.6%		66.1%	
3–7	47	45.3%		39.4%	
**Underlying COPD**			0.459		0.911
No	51	53.9%		48.8%	
Yes	12	40.9%		26.0%	
**Histology**			0.030		0.004
SQ	41	40.0%		36.4%	
Non-SQ	22	71.0%		68.7%	
**Pneumonectomy/bilobectomy**			0.540		0.760
No	56	51.4%		45.7%	

Yes	7	53.3%		51.4%	
**FEV1 at re-RT**			0.064		0.310
<1.5 L	9	100%		29.6%	
≥1.5 L	32	47.3%		52.2%	
**DLCO at re-RT**			0.751		0.004
<60%	10	72.0%		38.9%	
≥60%	23	53.5%		74.6%	
**Clinical stage at re-RT**			0.150		0.984
I-II	29	36.3%		48.1%	
II-IV	34	61.1%		44.9%	
**Recurrence type**			0.113		0.240
In-field	38	39.5%		44.3%	
Marginal	25	62.9%		49.6%	
**Recurrence at re-RT**			0.402		0.380
1st	56	48.4%		47.8%	
2nd or more	7	68.6%		40.0%	
**Time interval between two RT course**					0.290
<16 months	35	40.6%	0.056	40.9%	
≥16 months	28	65.3%		55.6%	
**CCRT at re-RT**			0.409		0.774
No	42	46.4%		44.2%	
Yes	21	60.0%		51.3%	
**Re-RT technique**			0.757		0.541
XRT	41	50.1%		44.1%	
PBT	22	55.1%		55.3%	
**BED_{10} at re-RT**			0.729		0.414
<80 Gy	32	54.1%		39.6%	
≥80 Gy	31	48.8%		56.2%	
**rCTV**			0.593		0.037
<75 cc	42	54.1%		54.8%	
≥75 cc	21	42.7%		31.2%	
**Cumulative BED_{10}**			0.933		0.926
≤160 Gy	37	52.1%		46.0%	
>160 Gy	26	51.5%		49.5%	
**Target – trachea location**			0.563		0.563
Non-ultracentral	43	45.8%		45.8%	
Ultracentral	20	62.7%		62.7%	
**Target – bronchus location**			0.877		0.877
Non-ultracentral	43	49.6%		49.6%	
Ultracentral	20	55.6%		55.6%	
**Target – esophagus location**			0.868		0.868
Non-ultracentral	52	50.4%		50.4%	
Ultracentral	11	53.6%		53.6%	

Abbreviations: RT, radiotherapy; SQ, squamous cell carcinoma; CCI, Charlson comorbidity index; COPD, chronic obstructive pulmonary disease; ECOG, Eastern Cooperative Oncology Group performance status; FEV1, the first second of forced expiration; DLCO, diffusing capacity of lung for carbon monoxide; CCRT, concurrent chemoradiation therapy; rCTV, clinical target volume at re-RT; BED_{10}, biologically effective dose with alpha-beta ratio of 10 Gy

Table S4. Pulmonary and esophageal toxicities according to patient characteristics and dosimetric parameters

	Lung			Esophagus		
**Variables**	Grades 0–2	Grades 3–5	p-value	Grades 0–2	Grades 3–5	p-value
**Age at re-RT**			0.894			0.411
<65 years	30 (90.9%)	3 (9.1%)		29 (87.9%)	4 (12.1%)	
≥65 years	26 (86.7%)	4 (13.3%)		29 (96.7%)	1 (3.3%)	
**Sex**			0.723			0.934
Female	7 (100%)	-		7 (100%)	-	
Male	49 (87.5%)	7 (12.5%)		51 (91.1%)	5 (8.9%)	
**Smoking**			0.446			1.000
Non-smoker	11 (100%)	-		10 (90.9%)	1 (9.1%)	
Current or Ex-smoker	45 (86.5%)	7 (13.5%)		48 (92.3%)	4 (7.7%)	
**ECOG at re-RT**			0.805			1.000
0–1	53 (88.3%)	7 (11.7%)		55 (91.7%)	5 (8.3%)	
2	3 (100%)	-		3 (100%)	-	
**CCI**			0.798			1.000
1–2	15 (93.8%)	1 (6.3%)		15 (93.8%)	1 (6.2%)	
3–7	41 (87.2%)	6 (12.8%)		43 (91.5%)	4 (8.5%)	
**Underlying COPD**			1.000			0.591
No	45 (88.2%)	6 (11.8%)		46 (90.2%)	5 (9.8%)	
Yes	11 (91.7%)	1 (8.3%)		12 (100%)	-	
**Histology**			0.963			1.000
SQ	37 (90.2%)	4 (9.8%)		38 (92.7%)	3 (7.3%)	
Non-SQ	19 (86.4%)	3 (13.6%)		20 (90.9%)	2 (9.1%)	
**Pneumonectomy/bilobectomy**			1.000			0.161
No	50 (89.3%)	6 (10.7%)		53 (94.6%)	3 (5.4%)	
Yes	6 (85.7%)	1 (14.3%)		5 (71.4%)	2 (28.6%)	
**FEV1 at re-RT**			1.000			1.000
<1.5 L	16 (84.2%)	3 (15.8%)		18 (94.7%)	1 (5.3%)	
≥1.5 L	21 (95.5%)	1 (4.5%)		18 (81.8%)	4 (18.2%)	
**DLCO at re-RT**			0.738			1.000
<60%	8 (80.0%)	2 (20.0%)		9 (90.0%)	1 (10.0%)	
≥60%	21 (91.3%)	2 (8.7%)		21 (91.3%)	2 (8.7%)	
**Clinical stage at re-RT**			1.000			0.454
I–II	26 (89.7%)	3 (10.3%)		28 (96.6%)	1 (3.4%)	
II–IV	30 (88.2%)	4 (11.8%)		30 (88.2%)	4 (11.8%)	
**Recurrence type**			0.820			0.645
In-field	33 (86.8%)	5 (13.2%)		34 (58.6%)	4 (80.0%)	
Marginal	23 (92%)	2 (8.0%)		24 (41.4%)	1 (20.0%)	
**Recurrence at re-RT**			0.723			0.934
1st	49 (87.5%)	7 (12.5%)		51 (91.1%)	5 (8.9%)	
2nd or more	7 (100%)	-		7 (100%)	-	
**Time interval between two RT course**			0.448			0.794
<16 months	30 (85.7%)	5 (14.3%)		33 (94.3%)	2 (5.7%)	
≥16 months	26 (92.9%)	2 (7.1%)		25 (89.3%)	3 (10.7%)	
**Target – trachea location**			0.534			1.000
Non-ultracentral	37 (86.0%)	6 (14.0%)		40 (93.0%)	3 (7.0%)	
Ultracentral	19 (95.0%)	1 (5.0%)		18 (90.0%)	2 (10.0%)	
**Target – bronchus location**			0.271			0.055
Non-ultracentral	40 (93.0%)	3 (7.0%)		42 (97.7%)	1 (2.3%)	
Ultracentral	16 (80.0%)	4 (20.0%)		16 (80.0%)	4 (20.0%)	
**Target – esophagus location**			1.000			<0.001
Non-ultracentral	46 (88.5%)	6 (11.5%)		52 (100%)	-	
Ultracentral	10 (90.9%)	1 (9.1%)		6 (54.5%)	5 (45.5%)	
**CCRT at re-RT**			0.119			0.070
No	35 (83.3%)	7 (16.7%)		41 (97.6%)	1 (2.4%)	
Yes	21 (100%)	-		17 (81.0%)	4 (19.0%)	
**Re-RT technique**			0.427			0.194

PBT	21 (95.5%)	1 (4.5%)		21 (95.5%)	1 (4.5%)	0.810
XRT	35 (85.4%)	6 (14.6%)		37 (90.2%)	4 (9.8%)	
**rCTV, cc**	81.4 ± 158.6	97.0 ± 92.3	0.801	87.3 ± 157.7	34.6 ± 41.4	0.074
**BED$_{10}$ at re-RT, Gy**	85.3 ± 16.3	100.3 ± 34.1	0.292	87.6 ± 19.9	79.9 ± 2.3	0.009
**Re-RT fractions**	22.0 ± 9.5	14.0 ± 7.2	0.002	20.3 ± 9.4	30.8 ± 6.1	0.017
**Re-RT fraction size, Gy**	3.8 ± 3.0	6.7 ± 5.7	0.234	4.3 ± 3.5	2.2 ± 4.5	<0.001
**Cumulative BED$_{10}$, Gy**	164.4 ± 31.8	175.4 ± 28.6	0.387	167.6 ± 31.9	143.3 ± 11.7	0.098
**Lung, Dmean, Gy**	4.5 ± 2.9	6.4 ± 4.1	0.115			
**Lung, V20, %**	7.5 ± 5.4	10.4 ± 8.1	0.220			
**Lung, V5, %**	19.0 ± 13.0	24.7 ± 14.1	0.283			
**Lung, cumulative Dmean , Gy**	14.1 ± 6.2	15.5 ± 4.8	0.682			
**Lung, cumulative V20, %**	22.8 ± 12.2	24.8 ± 9.5	0.635			
**Lung, cumulative V5, %**	46.6 ± 19.3	50.3 ± 18.3	0.865			
**Esophagus, Dmax, Gy**				41.7 ± 23.8	67.2 ± 2.5	<0.001
**Esophagus, Dmean, Gy**				8.6 ± 8.1	19.0 ± 7.4	0.008
**Esophagus, V50, %**				4.8 ± 9.7	21.8 ± 10.7	<0.001
**Esophagus, cumulative Dmax, Gy**				83.6 ± 33.3	123.5 ± 10.4	<0.001
**Esophagus, cumulative Dmean, Gy**				25.0 ± 14.4	42.2 ± 11.3	0.012
**Esophagus, cumulative V50, %**				22.3 ± 19.4	39.4 ± 9.2	0.057

Abbreviations: RT, radiotherapy; SQ, squamous cell carcinoma; CCI, Charlson comorbidity index; COPD, chronic obstructive pulmonary disease; ECOG, Eastern Cooperative Oncology Group performance status; FEV1, the first second of forced expiration; DLCO, diffusing capacity of lung for carbon monoxide; CCRT, concurrent chemoradiation therapy; rCTV, clinical target volume at re-RT; BED$_{10}$, biologically effective dose with alpha-beta ratio of 10 Gy; 3D, three-dimensional conformal radiation therapy; IMRT, intensity-modulated radiotherapy; PBT, proton beam therapy; SBRT, stereotactic body radiotherapy
